# Use of the universal pain assessment tool for evaluating pain associated with TMD in youngsters with an intellectual disability

**DOI:** 10.4317/medoral.21584

**Published:** 2016-12-06

**Authors:** Giorgi* Dugashvili, Linda* Van den Berghe, Giorgi Menabde, Marina Janelidze, Luc Marks

**Affiliations:** 1 Centre of Special Care in dentistry, PaeCoMeDiS, Gent University Hospital, Gent, Belgium; 2 Ilia State University, Institute of Medical Research, Tbilisi, Georgia

## Abstract

**Background:**

The Universal Pain Assessment Tool (UPAT) was used to assess the level of pain in people with limited communication skills. The UPAT enables clinicians to consult a specialized pain management team more often and lead to earlier interventions. The purpose of this study was to determine, whether the UPAT could be used as an extra tool to collect data on functional TMJ pain and to assess orofacial pain levels related to temporomandibular disorder(s) (TMD) in people with intellectual disabilities (ID).

**Material and Methods:**

Non-down syndrome ID Athletes were screened during the Special Olympics European games in 2014. The clinical scores of possible functional jaw pain were collected using the UPAT, to indicate pain severity on a visual scale during different jaw movements (opening, closing and lateral).

**Results:**

Two hundred and four youngsters were screened by calibrated dentists. The majority (65%) of participants were male (133 male and 71 female athletes); age distribution ranged from 15 to 23 years (mean 19.25 ± 2.53). The results of the UPAT have shown the existence of functional TMJ pain in 32% (n=65) of the athletes without significant prevalence (*P* > 0.05) in this survey group.

**Conclusions:**

According to the results of the present study, the UPAT demonstrated that it could be a useful tool to detect the existence of functional jaw pain possibly associated with TMD and also a valid instrument to score pain intensity associated with TMD in people with ID.

**Key words:**Universal pain assessment tool - TMD in ID - TMD in youngsters.

## Introduction

Temporomandibular disorder(s) (TMD) is a general term that includes a group of clinical entities that affect the Temporomandibular Joint, the Masticatory Musculature and the Associated Structures. There are many different pathologies, both articular and muscular that can be included in this group. TMD are one of the most common causes of orofacial pain after dental pain (1). It is considered to be one of the 4 major symptom complexes in chronic orofacial pain, along with burning mouth syndrome, atypical facial pain and atypical odontalgia (2).  

Intelectual disability (ID) is defined as an impairment in the areas of development or cognitive activities. It is characterized by significant limitations both in intellectual functioning and in adaptive behaviour, which covers many everyday social and practical skills. This disability originates before the age of 18 (3). In people with ID, typical indicators, such as crying, grimacing, elevated blood pressure, or tachycardia, may be absent due to central nervous system (CNS) damage accompanying the ID. As a result, it is difficult to ascertain, if the person is in pain. In addition, some people with IDs exhibit self-injurious behaviours (4), which some professionals may mistakenly interpret as insensitivity to pain. In fact, these inappropriate behaviours may be a response to pain. Early assessment and appropriate intervention for pain enhances quality of life. Unfortunately, people with ID are less likely to see healthcare practitioners regularly or have their pain recognized and treated promptly (5).

Universal pain screening with a 0-10 pain intensity numeric rating scale (NRS) has been widely implemented in primary care medicine. Various pain assessment tools, such as Wong-Baker Faces Pain Rating Scale (6) the Poker Chip Tool (7), the Eland Colour Scale (8) and FLACC scale (9) have been used or adapted by clinicians.

As pain is a highly subjective and individualized, self-report is frequently cited as the gold standard of pain assessment and it should always be initially attempted, as it is the most reliable report of pain (10).

For clinicians, the challenge has been in how to obtain a valid and reliable assessment of pain from persons who are unable to provide a self-report. Given the fact, that non-communicating ID people are not usually able to use any self-rating scales, FLACC scales are used by nurses and physicians.

In the present study the Universal Pain Assessment Tool (UPAT) (11) was used among Special Olympics Athletes. As the Tool is an adapted version of the Wong-Baker Faces Pain Rating Scale, it would help assess pain according to their individual needs. The UPAT was believed to provide the opportunity to assess pain levels using faces or behavioural observations to interpret pain, when athletes are unable to communicate the intensity of their pain.

The positive feedback of this tool could enable clinicians in the early recognition of pain-related behaviour to avoid undertreatment in people with intellectual disabilities (12). Moreover, it would give the opportunity for earlier consultation of a special care management team in case of suspected pain (13).

The purpose of this study was to determine, whether the UPAT could be used as a valid instrument to assess orofacial pain levels related to TMD in people with ID.

## Material and Methods

Non-down syndrome athletes were screened during the Special Olympics 2014 European games. The athletes were invited to the “Special Olympics Special Smiles” site on a voluntary basis. Written consent was obtained from the athlete and a parent or guardian. In full accordance of the World Medical Association Declaration of Helsinki, the Joint Ethical Committee of the Ghent University Hospital approved the study as 2013/816.

**Figure 1:**
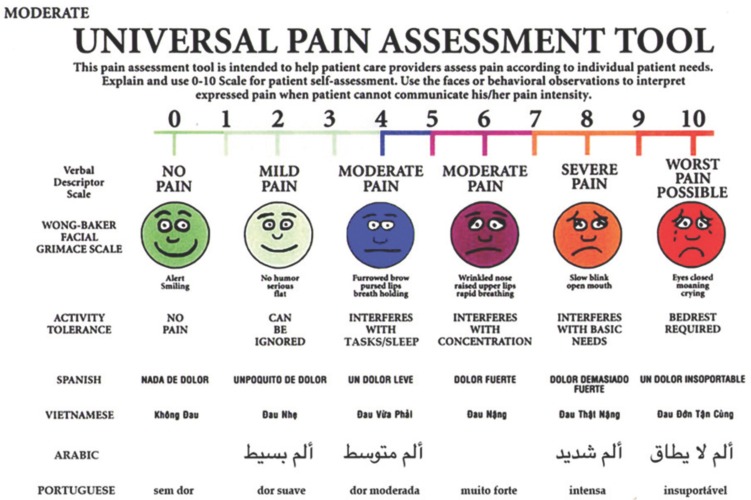
The Universal Pain Assessment Tool (UPAT), that has been used to identify functional TMJ pain.

Identification of functional jaw pain was measured using the UPAT (14) (Fig. 1). Five jaw movements were assessed (15): opening, maximum unassisted opening, maximum assisted opening (when moderate digital pressure was used to increase the degree of opening, if possible), left lateral excursion and right lateral excursion. The TMJ was palpated at 3 locations: lateral pole, posterior attachment (via the external acoustic meatus), and dorsal aspect (with 25-30 mm of jaw opening) (14). For right and left excursive movements, athletes were directed to open slightly and move their jaws as far as possible towards the right and left, even if it was painful, and then move their jaw back to a comfortable position and position their posterior teeth completely together each time. If the subject was confused about which direction they should move their jaw, they would be told to move their jaw towards the hand touched on the side of the desired movement. For all excursive movements, the subject was asked to repeat the movement three times.

If pain was reported during any of those movements, athletes were asked to indicate the severity of their pain on the UPAT. At no time during the screening, was a suggestion made nor was the subject led to respond about the presence of pain.

Joint noises (click, crepitus) were detected while placing fingers over the TMJ on either the right or the left side during opening and closing movements.

- Statistical analysis 

All of the data were recorded using the UPAT and processed by SPSS software (IBM® SPSS® Statistics 22 Version 22.0.0.1). The level of significance was set at 0.05.

## Results

**Figure 2:**
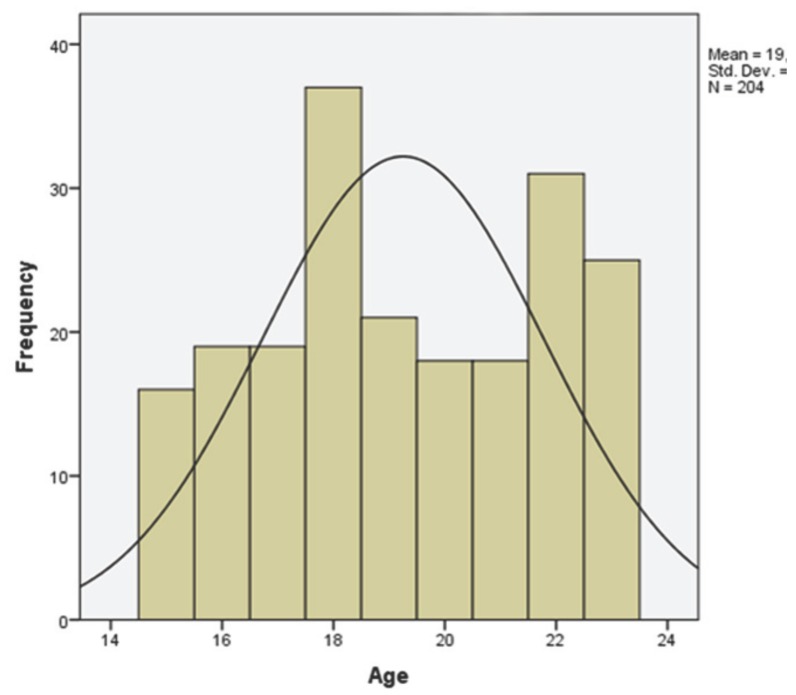
Age distribution of screened athletes.

Two hundred and four ID athletes were evaluated. The majority (65%) of participants were male (133 male and 71 female patients); age distribution ranged from 15 to 23 years (mean 19.25 ± 2.53) (Fig. 2). The results of the UPAT demonstrated the existence of functional TMJ pain in 32% (n=65) of the athletes without significant prevalence (*P* > 0.05) in the survey group.

**Figure 3:**
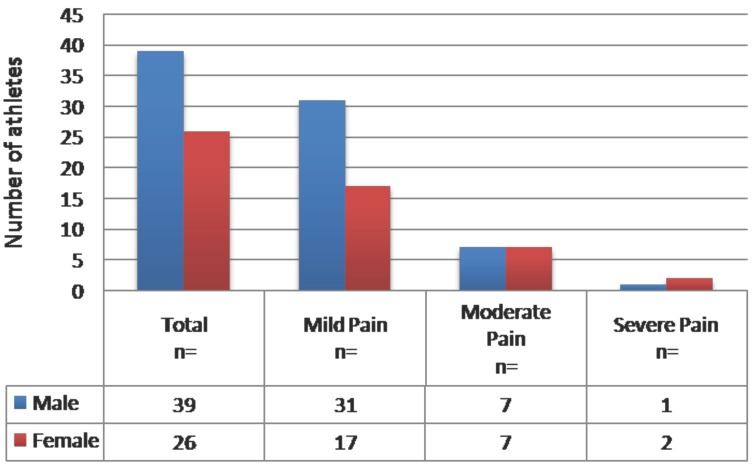
Distribution of pain levels among male and female athletes.

Seventy four percent of pain associated with TMD, was reported as mild. As the severity of pain level increased, the distribution of pain decreased, this can be markedly seen on figure 3.

**Figure 4:**
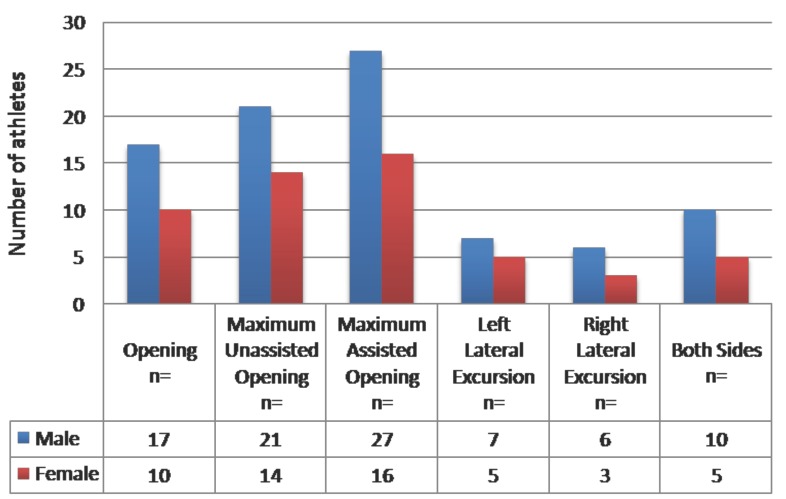
Distribution of functional jaw pain according to the different jaw movements.

Considering different jaw movements, the subjects reported far more pain on maximum opening without significant difference between assisted or unassisted opening (*P* > 0.05) (Fig. 4).

**Figure 5:**
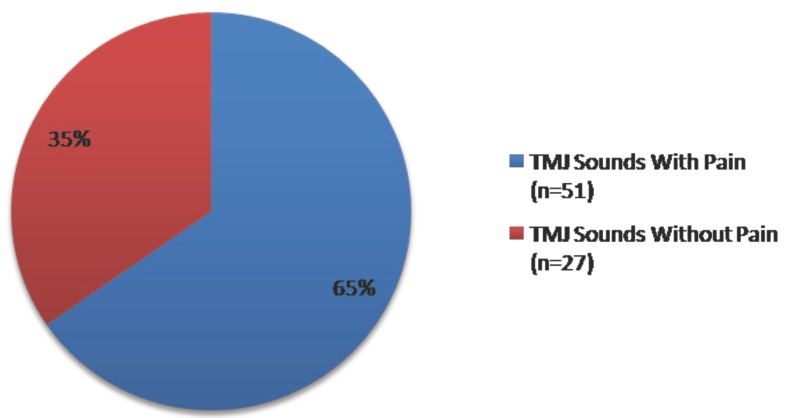
Distribution of TMJ sounds with and without functional pain report.

Joint sounds were found in 38% of subjects, 65% of these athletes also reported functional pain (Fig. 5).

## Discussion

There have been very limited studies and little information reported regarding TMD in people with ID (16). The selection of the study sample was defective as it included all “non-Down Syndrome ID athletes”. There are a great variety of IDs with many different clinical presentations that may cause a distortion in the validity of the results.

The present study has been conducted, in order to collect data on functional TMJ pain and to assess pain levels in people with ID, using the UPAT. Moreover, the present method for evaluating pain, would give opportunities to an earlier intervention in case of suspected pain.

Certain aspects can be considered to be the limitations of the present study. There is an unequal female/male ratio. This ratio is considered to be an important factor, because studies have demonstrated, that starting from late adolescence, prevalence of temporomandibular symptoms is higher in females than in males (17,18). Secondly, as far as only a particular group of people with ID has been studied, the results of the present research cannot be generalized. The outcomes have shown, that the issue is problematic and needs further study.

According to Kim *et al.* (19), TMD can occur at any age. They investigated various age groups and demonstrated that patients younger than 40 years of age have shown high proposition (74%), of which 38% is the youngest group (under 25 years). The study also suggested the tendency in younger patients needs to be continuously investigated.

On the contrary to the above-mentioned study, present research has shown a non-significant prevalence (*P* > 0.05) of pain associated with TMD in people with ID, within the ages of 15 to 23 years.

According to the same study (19), there is a tendency of female patients to be predisposed to TMD more than male patients. A possible explanation of this has been associated with thefemale sex hormone estrogen (20). In animal models estrogen has been shown to modulate inflammation in the TMJ (21,22). Moreover, Flake *et al.* (22) suggest, that testosterone and estrogen have opposing actions on TMJ. This could also account for the higher prevalence and severity of pain associated with TMD in females by suggesting that testosterone may mitigate, while estrogen exacerbates TMJ damage, particularly in the presence of overt inflammation.

The purpose of the present study was not to consider gender differences in pain prevalence, but a tendency towards absence of this differentiation could be observed. A possible explanation for this is the fact, that a population was screened rather than a patient group.

Authors in the present study palpated only the TMJ but not the superficial masticatory muscles (Masseter & Temporalis), which are the main source of extra-oral pain. Besides, palpation was done without any objective measurement of the force applied. Another limitation of the current study is that it analyzed TMJ signs and not real articular or muscular disorders according to the diagnostic criteria of the AAOP (15). A single sign from the masticatory system is not sinonymous with a TMD, nor does it automatically lead to the diagnosis of a TMD. Pain on palpation, joint noises and others are clinical signs that are frequently found in the examination of normal population without leading to a diagnosis of TMD. Consistent implementation of the DC/TMD in future studies will enable a more reliable definition of TMD cases for comparison of their findings. A questionnaire, in which the subjects are asked about pain during mastication would give additional benefits although the outcome could be questioned in this ID group.

The present study has outlined, that when pain associated with TMJ noises is present, TMD can be present. Similar findings can be found in the study of Ohrbach *et al.* (14). However, noises alone are no longer considered to be diagnostic for TMD, since they can be detected in a high proportion in general populations (23).

According to Glaros *et al.* (24) the existence of stress and emotional distress, together with oral parafunctional behaviours can be predictive for TMD. They suggest that treatment focussing on coping with psycho-emotional problems could be effective by diminishing excessive masticatory muscle tension. Myogenous TMD is frequently considered to be the most common type of TMD and is generally caused by increased muscle activity triggered by emotional stress (25). As far as the TMJ screening was performed during the Special Olympics event, when the athletes were taking part in the sports competition, the stress factor should be taken into consideration while interpreting the results.

According to Chisnoiu *et al.* (26), stress and anxiety issues can influence on the person’s psyche and may lead to pain, due to spasms of the internal, external pterygoid and masseter muscles caused by bruxism (grinding, clenching, bracing). In conformity with the abovementioned, TMD in ID athletes could be considered as stress related. However, the issue needs to be further researched and cannot be generalized in people with ID.

## Conclusions

Although TMD is not considered to be life-threatening, it can be deleterious to the quality of life (27) for people with ID, as their symptoms could become chronic and difficult to manage if they are not detected and eliminated at an early stage.

According to the results found in this study, the UPAT demonstrated to be a an additional tool to detect the existence of functional jaw pain possibly associated with TMD.

If UPAT could be implemented in general practice of dentistry for evaluating pain associated with TMD in people with special needs, further research will be needed to determine whether screening for pain will improve patient outcomes.

As far as there are very limited studies reported in the literature regarding TMD in people with ID, the present study could be the initiative for future research in this field.
